# Postischemic Neuroprotection of Aminoethoxydiphenyl Borate Associates Shortening of Peri-Infarct Depolarizations

**DOI:** 10.3390/ijms23137449

**Published:** 2022-07-04

**Authors:** Rocío Fernández-Serra, Emma Martínez-Alonso, Alberto Alcázar, Mourad Chioua, José Marco-Contelles, Ricardo Martínez-Murillo, Milagros Ramos, Gustavo V. Guinea, Daniel González-Nieto

**Affiliations:** 1 Center for Biomedical Technology, Universidad Politécnica de Madrid, 28223 Madrid, Spain; rocio.fernandez@ctb.upm.es (R.F.-S.); milagros.ramos@ctb.upm.es (M.R.); gustavovictor.guinea@ctb.upm.es (G.V.G.); 2 Departamento de Tecnología Fotónica y Bioingeniería, ETSI Telecomunicaciones, Universidad Politécnica de Madrid, 28040 Madrid, Spain; 3 Departamento de Ciencia de Materiales, ETSI Caminos, Canales y Puertos, Universidad Politécnica de Madrid, 28040 Madrid, Spain; 4 Silk Biomed SL, 28260 Madrid, Spain; 5 Department of Research, Hospital Universitario Ramón y Cajal, IRYCIS, 28034 Madrid, Spain; emma.martinez@hrc.es (E.M.-A.); alberto.alcazar@hrc.es (A.A.); 6 Laboratory of Medicinal Chemistry, Institute of General Organic Chemistry (CSIC), 28006 Madrid, Spain; mchioua@gmail.com (M.C.); jlmarco@iqog.csic.es (J.M.-C.); 7 Department of Translational Neuroscience, Instituto Cajal (CSIC), 28002 Madrid, Spain; r.martinez@cajal.csic.es; 8 Biomedical Research Networking Center in Bioengineering Biomaterials and Nanomedicine (CIBER-BBN), 28029 Madrid, Spain; 9 Biomaterials and Regenerative Medicine Group, Instituto de Investigación Sanitaria del Hospital Clínico San Carlos (IdISSC), 28040 Madrid, Spain

**Keywords:** 2-APB, cholesteronitrone F2, neuroprotection, spreading depolarization, peri-infarct depolarizations, hypoperfusion, oxidative stress, stroke

## Abstract

Brain stroke is a highly prevalent pathology and a main cause of disability among older adults. If not promptly treated with recanalization therapies, primary and secondary mechanisms of injury contribute to an increase in the lesion, enhancing neurological deficits. Targeting excitotoxicity and oxidative stress are very promising approaches, but only a few compounds have reached the clinic with relatively good positive outcomes. The exploration of novel targets might overcome the lack of clinical translation of previous efficient preclinical neuroprotective treatments. In this study, we examined the neuroprotective properties of 2-aminoethoxydiphenyl borate (2-APB), a molecule that interferes with intracellular calcium dynamics by the antagonization of several channels and receptors. In a permanent model of cerebral ischemia, we showed that 2-APB reduces the extent of the damage and preserves the functionality of the cortical territory, as evaluated by somatosensory evoked potentials (SSEPs). While in this permanent ischemia model, the neuroprotective effect exerted by the antioxidant scavenger cholesteronitrone F2 was associated with a reduction in reactive oxygen species (ROS) and better neuronal survival in the penumbra, 2-APB did not modify the inflammatory response or decrease the content of ROS and was mostly associated with a shortening of peri-infarct depolarizations, which translated into better cerebral blood perfusion in the penumbra. Our study highlights the potential of 2-APB to target spreading depolarization events and their associated inverse hemodynamic changes, which mainly contribute to extension of the area of lesion in cerebrovascular pathologies.

## 1. Introduction

Ischemic stroke is a leading cause of death and disability worldwide. Approximately 5.5 million people die from stroke every year, and half of the survivors remain chronically disabled. In 2013, stroke was responsible for 113 million disability-adjusted life years [1]. Due to the demographic changes, these numbers are predicted to increase. In Europe, by 2025, there will be approximately 1.5 million new stroke cases per year [2].

It has been estimated that about 90% of the stroke burden can be imputable to modifiable risks factors such as hypertension, smoking, obesity, physical activity, diabetes, or dyslipidemia [3]. Blood pressure is, for example, the most determinant risk factor for stroke. Hypertension has been reported in almost 70% of stroke patients and there is strong evidence that antihypertensive therapy prevents stroke events and reduces mortality, especially in the middle-aged population [4]. Epidemiological studies indicate the complex interaction between genetic and environmental risks factors over the development of atherosclerotic disease. Among these factors, hypertension is the main cause of atherosclerosis and carotid artery disease, which is responsible for around 20% of all cases of ischemic stroke [5]. In contrast, atrial fibrillation (AF) is a major cause of large vessel occlusions and another risk factor for stroke, especially in the elderly population. This common type of cardiac arrhythmia has been found in 10–25% of stroke cases, and 40% of stroke cases in patients above 80 years [6]. Although in many cases AF has an uncertain origin, anticoagulation therapies and risk-modifying strategies such as antihypertensive treatment, smoking cessation, or physical activity have reduced the risk of stroke in patients with AF [6]. Several pieces of evidence support the association of AF with relatively treatable underlying pathologies and conditions. For example, hyperthyroidism has been linked with AF [7], probably through the shortening of cardiac action potentials [8]. There is still debate about whether hyperthyroidism treatment prevents or reverses AF [9]. AF is also the most common atrial arrhythmia found in Brugada syndrome [10], an inherited channelopathy caused by mutations in the sodium (Na^+^) and calcium (Ca^2+^) channels in around 30% of patients [11]. In addition, patients with long QT syndrome type 3 (LQT3) have also been associated with a high risk of early onset of AF while LQT2 patients were apparently protected against AF [12]. In contrast, although moderate/high physical activity has been associated with lower risks of stroke incidence [13,14], vigorous physical activity has been linked with high risk of AF [15].

Together with the control of intracranial pressure and glycemia levels, primary care for ischemic stroke is based on pharmacological and surgical recanalization. Nevertheless, few patients are eligible for recanalization due to the high risk of hemorrhagic transformation. In addition, a significant fraction of patients are refractory to these treatments [16]. To reduce mortality and disability rates, neuroprotective strategies against secondary damage have been investigated for decades. Targeting excitotoxicity and oxidative stress are very promising approaches, but only a few compounds have reached the clinic with relatively good positive outcomes [17,18]. This has been the case for edaravone, a medical compound used in Japan and China that has not been approved for clinical use in Europe or America. The search for new molecules and targets to attenuate the consequences of brain ischemia is still a priority.

Oxygen deficiency impairs Na^+^/K^+^ ATPase function, leading to neuronal depolarization and an increase in the intracellular Ca^2+^ concentration, which triggers the release of excitatory neurotransmitters, activation of NMDA receptors, and Ca^2+^ entry in a vicious circle [19]. Although NMDA receptors mainly seem to be responsible for the increase in the Ca^2+^ concentration and neurotoxicity, most drugs that hypoactivate NMDA receptors have failed in the clinical context. Other sources of Ca^2+^ entry might tentatively contribute to enhanced excitotoxicity, oxidative damage, and neuronal death. 2-Aminoethoxydiphenyl borate (2-APB) is a potent antagonist of Ca^2+^ entry, which has been demonstrated to induce neuroprotection in different in vitro and in vivo models of brain ischemia-reperfusion [20]. In addition, 2-APB has also shown protective effects in other pathological contexts, a fact probably ascribed to the versatility of the effects exerted by this molecule on different channels and receptors. 2-APB prevented mitochondrial Ca^2+^ overload and cytochrome c release and provided liver protection after ischemia-reperfusion injury [21]. 2-APB treatment attenuated hydrogen peroxide (H_2_O_2_)-induced cell death by inhibiting intracellular Ca^2+^ influx and provided cardiomyocyte protection after ischemia-reperfusion [22]. In another study, 2-APB was protective after renal ischemia–reperfusion injury, preventing the accumulation of Ca^2+^ by blocking store-operated calcium channels in renal efferent arterioles [23].

After cerebral ischemia, the neuroprotective mechanisms of 2-APB were apparently linked with the inhibition of the transient receptor potential cation channel TRPM7, a channel that is overexpressed after brain ischemia and contributes to the increase in intracellular Ca^2+^, oxidative stress, and neuronal death [24]. In vitro, 2-APB protected neurons from death induced by oxygen-glucose deprivation and subsequent reoxygenation [25], an effect linked to the inhibition of TRMP2 channels that highlights the known unspecifity of 2-APB. A previous study also reported the neuroprotective effect of 2-APB in a transient ischemia model [26], which was related to the inhibition of thrombus formation and platelet pro-coagulant activity through the antagonization of Orai1 channels, which mediate store-operated Ca^2+^ entry [26]. Despite its unspecifity, 2-APB seems very promising in ischemia-reperfusion models. Since a significant fraction of stroke patients do not experience spontaneous reperfusion and/or are refractory or ineligible for recanalization therapies, in this study, we examined the possibility that 2-APB could also exert a potential benefit after prolonged ischemia. We tested the neuroprotective effect of 2-APB in a model of permanent ischemia in mice, which is a preferable model to mimic this specific stroke type [27].

## 2. Results

### 2.1. Evaluation of Infarct Size and Sensorimotor Coordination

**Figure 1:**
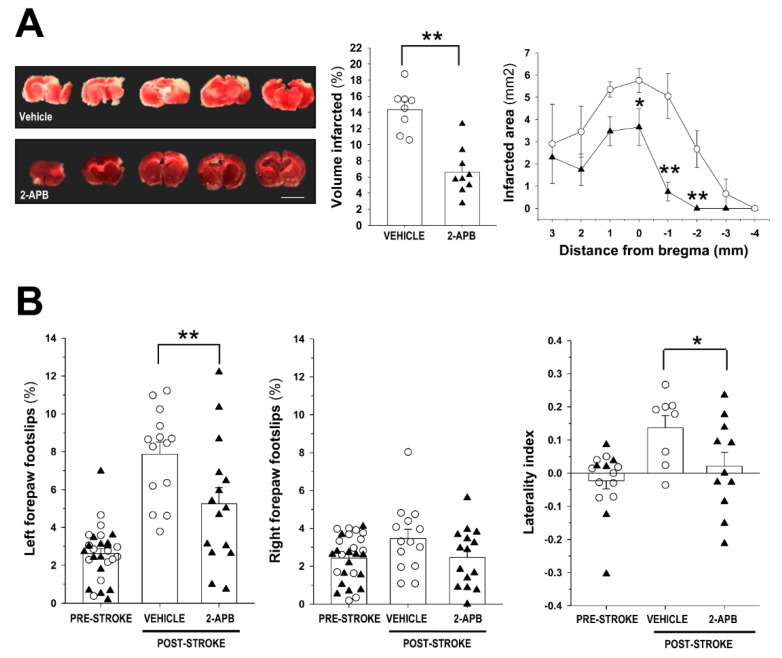
**2-APB reduces damage extension and improves poststroke behavioral performance after pMCAO.** (**A**) Left panel, representative 2,3,5-triphenyltetrazolium-stained (TTC) images of coronal sections 24 h after pMCAO in vehicle- and 2-APB-treated mice. The infarct area (in white) is mainly localized in somatosensory cortex areas (scale bar 0.5 cm). Middle panel, infarct volume in vehicle- and 2-APB-treated mice expressed as a percentage of the contralateral noninfarcted hemisphere volume. Right panel, infarcted area along the rostrocaudal axis as measured from bregma. A minimum of 8 mice per group, vehicle (circles) or 2-APB (triangles), were analyzed. (**B**) Percentage of foot faults with the left (left panel) and right (middle panel) forepaws in the grid-walking test before and one week after stroke. The right panel shows the laterality index (LI) related to asymmetry in the use of each forelimb. Preferential use of the unaffected forepaw (right) is denoted by higher positive values. A minimum of 8 mice per group, vehicle or 2-APB, were analyzed in every behavioral test. Circles and triangles represent untreated vehicle and 2-APB-treated mice, respectively. Data are represented as the means ± standard error of the mean (SEM). The asterisks denote significant differences between vehicle and 2-APB mice (Student’s t-test for infarct volume; two-way analysis of variance (ANOVA) for infarcted area across rostrocaudal axis and behavioral tests; * *p* < 0.05; ** *p* < 0.01).

We tested the possible neuroprotective effect of 2-APB after permanent middle cerebral artery occlusion (pMCAO). This model produced infarcts restricted to cortical areas, especially affecting the primary somatosensory cortex, while subcortical structures were less affected (Figure 1A). When administered acutely after pMCAO, 2-APB significantly decreased the total infarct volume and the infarct size across the rostro-caudal axis (Figure 1A). We analyzed the consequences of this effect at the behavioral level. In the grid-walking test, one week after stroke, this compound significantly reduced the sensorimotor deficits of the left forepaw (contralateral to the damaged hemisphere) (Figure 1B, left panel). The scores for the right forepaw (contralateral to the nondamaged hemisphere) did not differ between untreated vehicle and 2-APB-treated mice nor with respect to the prestroke condition (Figure 1B, middle panel). In the cylinder test, the untreated vehicle mice presented a preferential use of the right forepaw (contralateral to the noninfarcted hemisphere) and rarely used the left forepaw (contralateral to the infarcted hemisphere) while 2-APB-treated mice did not show preferential use of any forepaw, and the scores were not significantly different with respect to the prestroke condition (Figure 1B, right panel).

### 2.2. Assessment of Cortical Brain Function by Somatosensory Evoked Potentials

**Figure 2:**
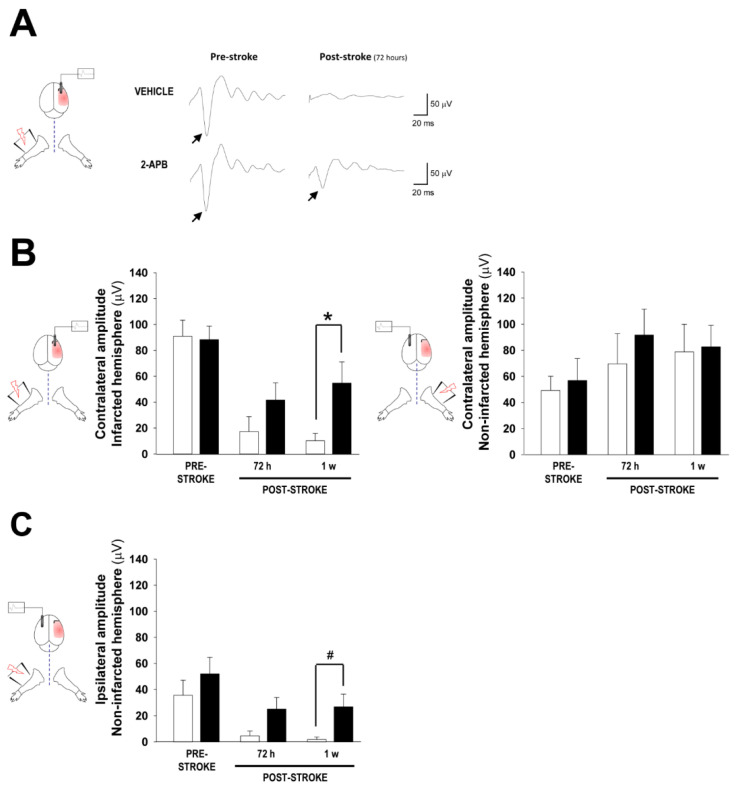
**Functional evaluation of the somatosensory cortex in 2-APB-treated stroke mice.** (**A**) Representative contralateral somatosensory evoked potentials (SSEPs) in response to left forepaw stimulation before and after ischemia in vehicle- and 2-APB-treated mice. The cartoon shows the relation between the stimulated forepaw (left) and the position of the recording electrode in the contralateral hemisphere (right). (**B**) Amplitude of contralateral SSEPs in the right (infarcted) and left hemispheres, respectively, under basal (pre-stroke) and post-stroke conditions (3 and 7 d) in vehicle and 2-APB mice. (**C**) Amplitude of ipsilateral SSEPs in the left hemisphere. In total, 7 and 11 mice were used for the vehicle and 2-APB groups, respectively. The white and black bars represent vehicle- and 2-APB-treated mice, respectively. The data are shown as the mean ± SEM. The asterisks denote significant differences between groups (two-way ANOVA; * *p* < 0.05; the symbol # denotes *p* values ranging between 0.1 and 0.05).

Controversial aspects regarding the relationship between infarct size and recovery in rodents have been reported since a reduction in infarct size does not always lead to behavioral improvement. In fact, specific behavioral tests can yield positive results that cannot be confirmed in alternative tests, and in many examples, the timeline of recovery might be quite different in every behavioral test applied [28]. We performed additional experimentation to examine whether the reduced damage area in 2-APB-treated mice translated into the preservation of function in cortical territories. This was assessed by the recording and analysis of somatosensory evoked potentials (SSEPs). Under the pre-stroke condition, a cortical field potential emerged in the primary somatosensory cortex of the contralateral hemisphere in response to forelimb stimulation (Figure 2A). After stroke, in the infarcted hemisphere, SSEPs were almost absent in untreated vehicle mice while significant cortical activity was still present in 2-APB-treated mice (Figure 2A,B, left panel). In striking contrast, the amplitude of contralateral responses in the noninfarcted hemisphere was similar between untreated vehicle and 2-APB-treated mice (Figure 2B, right panel). Cortical activity in the primary somatosensory cortex of the infarcted contralateral hemisphere remained present at later time points in 2-APB-treated mice, reaching the activity registered prior to the stroke condition (Supplementary Figure S1). In addition, ipsilateral activation in the non-damaged hemisphere was compatible with the contralateral response in the infarcted hemisphere (Figure 2C and Supplementary Figure S1). Among the different mechanisms, ipsilateral activation has mostly been related to the preactivation of the opposite hemisphere through interhemispheric connections [29,30]. In comparison with untreated vehicle mice, the 2-APB-treated mice showed higher contralateral responses in the infarcted hemisphere, which produced higher ipsilateral responses in the noninfarcted hemisphere. However, a negligible ipsilateral response was detected in the vehicle group, denoting the absence of previous activation in the contralateral infarcted hemisphere in this group of animals (compare Figure 2B left panel with Figure 2C). Collectively, 2-APB was neuroprotective after permanent ischemia. This molecule reduced the extent of the damage after pMCAO, preserving, at least partially, the functionality of the primary somatosensory cortex, which translated into better poststroke behavioral performance.

### 2.3. Evaluation of Secondary Injury: Oxidative Stress and Inflammatory Response

**Figure 3:**
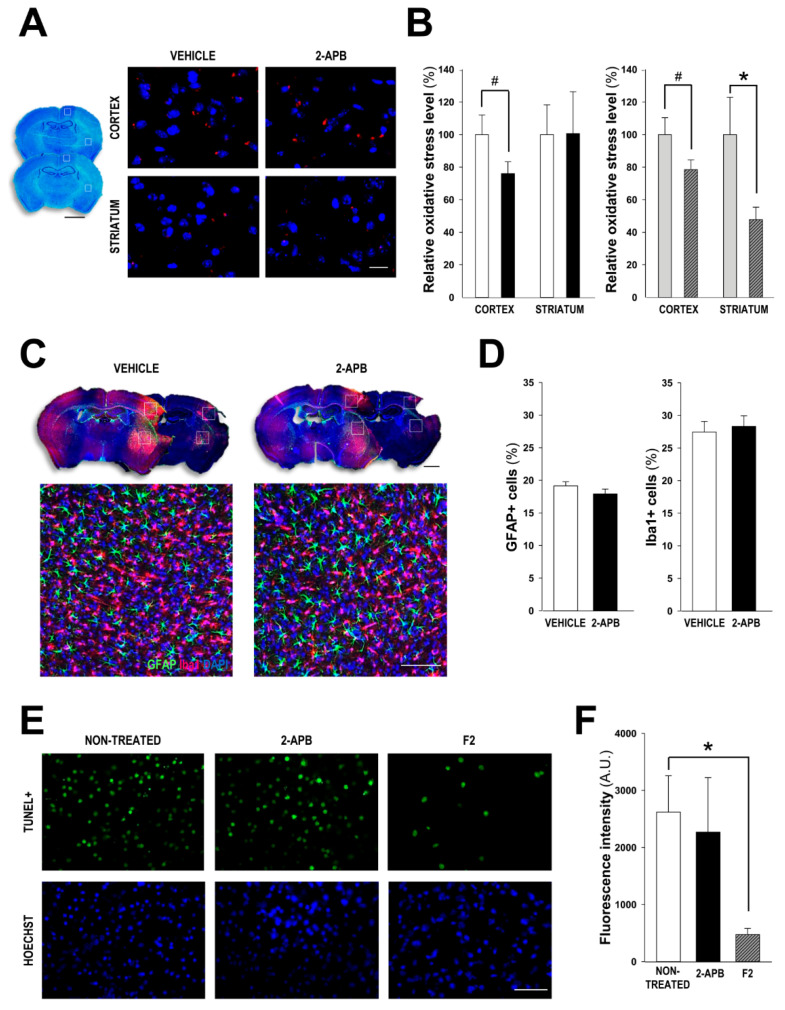
**2-APB does not modify oxidative stress, inflammation, or apoptotic neuronal death in mice submitted to pMCAO.** (**A**) Representative fluorescent microscopy images showing dihydroetidium-positive (DHE) fluorescence (pseudocolored red) in the perilesional cortex and striatum of the infarcted hemisphere. Nuclei were stained with DAPI dye (pseudocolored blue). Scale bar: 15 μm. On the left part, coronal brain sections show the areas that were analyzed (white squares). Scale bar: 2 mm. (**B**) Left, normalized oxidative content in the perilesional cortex and striatum in the infarcted hemisphere of untreated vehicle (white bars) and 2-APB (black bars) treated mice. A minimum of 27 coronal sections were analyzed from 8 mice per group. Right, normalized oxidative content in the infarcted hemisphere of untreated (grey bars) and cholesteronitrone F2, (dashed dark grey bars) treated mice. A minimum of 14 coronal sections were analyzed from 5 mice per group. (**C**) Representative fluorescent microscopy images showing GFAP-positive (pseudocolored green) and Iba1-positive (pseudocolored red) fluorescence. Nuclei were stained with Hoechst dye (pseudocolored blue). Scale bar: 100 μm. On the top, coronal brain sections show the areas that were analyzed (white squares). Scale bar: 1 mm. (**D**) Quantification of GFAP-positive and Iba1-positive cells in relation to the number of nuclei. A minimum of 4 images per brain were analyzed from 7 mice per group. (**E**) Representative images of apoptosis detection by Terminal deoxynucleotidyl transferase-mediated dUTP Nick-End Labeling-positive (TUNEL) assay and the corresponding Hoechst counterstaining in brain sections from the primary somatosensory cortex of non-treated, 2-APB-, and F2-treated stroke mice. TUNEL-positive neurons were visualized by fluorescence microscopy (green) in the primary somatosensory cortex. Scale bar: 50 μm. (**F**) The fluorescence intensity of the TUNEL signal was quantified and normalized to the number of nuclei in the primary somatosensory cortex. A minimum of 4 images per brain sample were analyzed from 3 (F2), 7 (vehicle), and 7 (2-APB) mice. Data are represented as the means ± SEM. The asterisks denote significant differences between groups (Student’s t-test for oxidative stress; Kruskal–Wallis test for TUNEL; * *p* < 0.05). The symbol # denotes *p* values ranging between 0.1 and 0.05).

As it has been shown that 2-APB interferes with intracellular Ca^2+^ dynamics by the antagonization of several channels and receptors [31], and because Ca^2+^ overload due to glutamate-induced neuronal toxicity may lead to mitochondrial dysfunction and oxidative stress, especially after reperfusion [32], we wondered whether the neuroprotective effect of this molecule could be associated with a possible reduction in oxidative stress, since prolonged ischemia without restoration of cerebral blood flow (CBF) can also produce an accumulation of reactive oxygen species [33]. To this end, we analyzed the in vivo content of superoxide anion (O_2_•−) in 2-APB-treated stroke mice. The treatment with 2-APB slightly reduced the content in O_2_•− in the perilesional cortex while in the peri-lesional striatum, no differences were observed in the oxidative stress levels between untreated and 2-APB-treated mice after pMCAO (Figure 3A,B-left). Due to the lack of an antioxidant effect of 2-APB, we wondered if other molecules with known antioxidant capacity could modify the content of reactive oxygen species in our model. With this aim, pMCAO mice were acutely treated with cholesteronitrone F2, a molecule that has been previously reported to exert antioxidant activity in transient and global ischemia models [34,35]. The treatment with F2 showed a reduction in the levels of oxidative stress after permanent ischemia (Figure 3B-right), denoting that the neuroprotection exerted by 2-APB might not be attributable to a possible antioxidant intracellular Ca^2+^-dependent mechanism.

The migration of inflammatory cells towards damaged areas takes place following ischemic insult. The generation of Ca^2+^ waves leads to the recruitment of microglia cells and activation of astrocytes [36]. However, the immunohistochemistry analysis of brain areas surrounding the damaged tissue did not show differences in the content of astrocytes (GFAP-positive cells) or immune cells (Iba1-positive macrophages/microglia) (Figure 3C,D, Supplementary Figure S2). Secondary damage in the penumbra leads to the apoptotic death of neurons. We wondered whether the neuroprotective effect of 2-APB linked with a decrease in the infarct volume was related to a reduction in apoptotic neuronal death. However, we did not detect differences in the content of apoptotic neurons in the primary somatosensory cortex between non-treated (vehicle) and 2-APB-treated mice, as assessed by TUNEL assay (Figure 3E,F). In contrast, the administration of cholesteronitrone F2 decreased the death of neurons via apoptosis after pMCAO (Figure 3E,F). These results prove that after permanent ischemia, 2-APB does not exert neuroprotection by means of a reduction in secondary damage mechanisms related to inflammation or the prevention of neuronal apoptosis.

### 2.4. Evaluation of Secondary Injury: Peri-Infarct Depolarizations and Cerebral Blood Flow

**Figure 4:**
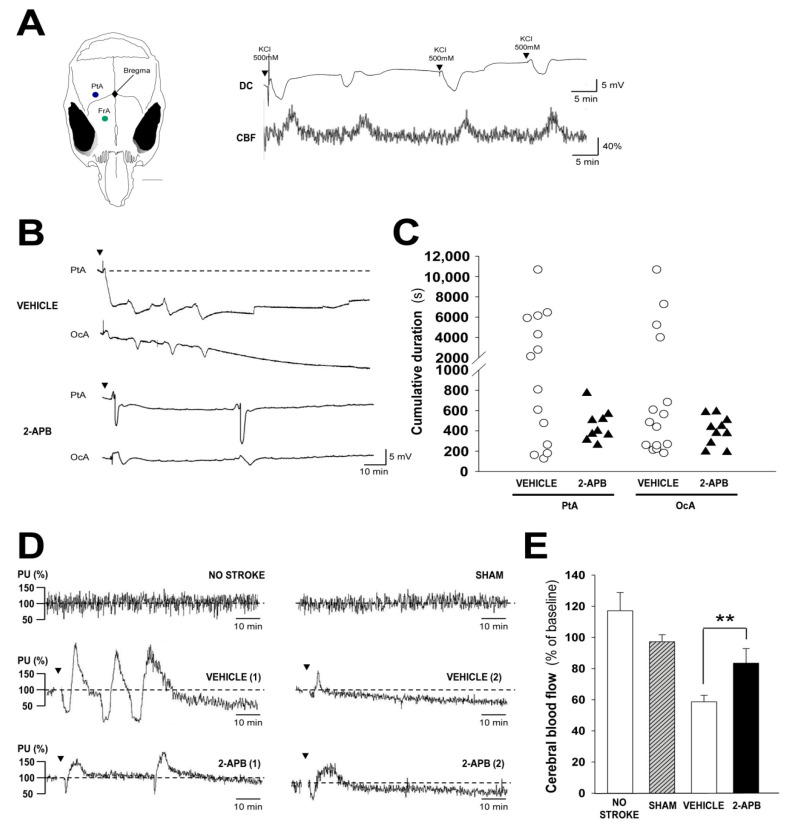
**Influence of 2-APB on the cortical depolarization events and cerebral blood flow (CBF) changes elicited by ischemia**. (**A**) Left, cartoon showing a dorsal view of a mouse skull illustrating the position of the DC recording electrode in the parietal cortex (PtA, blue circle) and the place for KCl application in the frontal cortex (FrA, green circle). Scale bar: 2 mm. Right, representative example of simultaneous recordings of DC signals and adjacent cerebral blood flow (CBF) changes induced by topic application of KCl (500 mM). For every negative DC deflection, a delayed increase in CBF was detected, a characteristic hallmark of KCl-induced depolarization in the intact (non-ischemic) brain. (**B**) Representative DC recordings from untreated and 2-APB-treated mice immediately after pMCAO. (**C**) Cumulative depolarization time in untreated vehicle or 2-APB-treated mice for 3 h after pMCAO obtained from parietal and occipital recordings. Each symbol in the graph represents the cumulative time of a single mouse. Data from 16 untreated vehicle and 11 2-APB-treated mice were analyzed. (**D**) Representative traces of CBF changes in non-stroke and sham mice (top) and immediately after pMCAO in untreated (middle) and 2-APB (bottom) treated mice. Note the existence of CBF transients composed of a fast hypoperfusion wave that was followed by a longer phase of hyperemia. (**E**) Quantification of residual CBF 100 min after stroke in untreated vehicle and 2-APB-treated mice. In non-stroke and sham animals, CBF was relatively stable during an equivalent recording period. Data from 3 (no-stroke), 5 (sham), 8 (vehicle), and 8 (2-APB) mice were analyzed for CBF determination. Data are represented as the means ± SEM. The asterisks denote significant differences between groups (one-way ANOVA ** *p* < 0.01).

After brain ischemia, a mechanism that contributes to secondary injury is cortical spreading depolarization (CSD) and its associated hemodynamic response that causes hypoperfusion, enlarging the infarct size [37,38,39,40]. Since CSD waves have been linked with increasing levels of intracellular Ca^2+^ in neurons [41,42], we examined the possible influence of 2-APB in the generation of CSD events after ischemia. Artificially, cortical depolarizations can be elicited in the non-ischemic brain by the direct application of KCl on the brain surface. The application of KCl in the frontal cortex produced regular negative voltage shifts in parietal locations (Figure 4A) that propagated to occipital areas (Supplementary Figure S3). A characteristic phase of hyperemia was observed for every CSD, although the increase in CBF outlasted the CSD events [43] (Figure 4A). In contrast, the cortical depolarizations waves elicited after ischemia were more complex and highly variable among different animals (Figure 4B). We analyzed the responses obtained from a total of 16 vehicle and 11 2-APB-treated mice after pMCAO. In some animals, pMCAO produced one single cortical depolarization change and no subsequent responses were observed during a recording interval time of 3 h post-stroke. In other cases, pMCAO induced two or more negative cortical depolarizations shifts spaced at irregular intervals with a variable amplitude and time duration (Figure 4B), and with a pattern of depolarization responses (specially the vehicle group) quite similar to the spontaneous transient negative potential shifts reported in rats after MCAO [37]. Collectively, the cortical depolarizations were generically longer in untreated mice than in 2-APB-treated animals (Figure 4C). However, the amplitude of cortical depolarizations in the parietal (vehicle: 8.5 mV ± 1.1; 2-APB: 9.6 mV ± 1.2, *p* = 0.51) and occipital (vehicle: 7.3 mV ± 1.8; 2-APB: 4.6 mV ±1.2, *p* = 0.55) cortices did not differ between the vehicle- and 2-APB-treated animals.

CSD events, peri-infarct depolarizations (PIDs) in ischemic tissue, have been associated with profound hemodynamic changes after ischemia. An increasing number of hypoperfusion episodes has been associated with a higher incidence of PIDs [40]. Thus, in another set of experiments, we measured changes in CBF in medial parietal (MP) areas that should correspond with a zone of transition between penumbra and non-affected tissue. We chose this location because in untreated mice, pMCAO causes an immediate but slight reduction in CBF in this area. In addition, 24 h after permanent ischemia, this location (MP) usually borders the infarct area in this specific mouse strain [44]. CBF was monitored for 100 min after stroke and animals showed in this location biphasic transients composed of a fast hypoperfusion phase followed by a prolonged hyperemia (Figure 4D). We did not detect significant differences in the number of these biphasic transients or in the magnitude of hyperemic events between vehicle- and 2-APB-treated mice (Supplementary Figure S4). However, together with these biphasic events, a gradual and slow hypoperfusion was observed in both groups of animals, although the residual CBF was significantly lower in untreated animals than in 2-APB-treated animals while sham or non-ischemic animals showed no significant variation of CBF (Figure 4D,E). Collectively, our results suggest a lower incidence of PID in 2-APB-treated animals that was linked with a higher CBF in the penumbra.

## 3. Discussion

Stroke continues to be a devastating and incurable pathology. The identification of new targets and therapeutic compounds is essential to overcome current limitations in the field and address this complex challenge. In this study, we tested the effect of 2-APB, a known modulator of Ca^2+^ signaling that has shown neuroprotective properties in models of transient brain ischemia. We described for the first time that, when administered early after stroke, 2-APB exerted a neuroprotective benefit after permanent ischemia, which resulted in decreased extension of damage and in behavioral improvement. Our study highlights the potential of 2-APB in this specific ischemic model, which reflects a very common clinical condition, because a significant fraction of stroke patients are not eligible or are refractory to pharmacological and/or surgical recanalization. Throughout the recordings of SSEPs, we demonstrated that 2-APB treatment translated into greater preservation of cortical activity compared to no treatment (vehicle) in stroke subjects. The neuroprotective effect of 2-APB did not seem to be associated with apparent changes in apoptotic neuronal content, inflammation, and oxidative stress. In contrast, 2-APB might exert its positive effects by decreasing the cumulative incidence of PIDs and partially decreasing the gradual hypoperfusion that occurs in penumbra regions and contributes to enlarge the infarct size [40]. This agrees with previous concepts, where a higher incidence of PIDs is associated with progressive lower residual CBF in the penumbra [40].

It has been described that after ischemia, depolarization waves propagate through the gray matter of the central nervous system and cause abrupt depolarization of neurons and loss of transmembrane ionic gradients [43]. These waves emerge from peri-infarcted tissue in patients and ischemia models [45,46] and concur with the swelling of neurons and the shrinkage of the extracellular space [47,48]. Neuronal damage is increased by prolonged spreading depolarization and the cumulative time of depolarization in the ischemic border zone correlates better with postischemic damage in both transient and permanent ischemia models [37,49]. The emergence of spreading depolarization events in peri-infarcted tissue is probably a consequence of the local increase in potassium and glutamate in penumbra areas bordering the infarct. Events of hypoperfusion are coincident with spreading depolarization induced by ischemia [40]. Thus, an increased cumulative depolarization time not only contributes to impaired metabolism and exacerbated energy deficits but also enlarges the area of the hypoperfused cortex in the penumbra and surrounding regions, increasing the size of the definitive lesion [40,50,51].

Spreading depression and PIDs are restricted to half of patients with acute brain injury [45], a fact probably associated with the stochastic nature of these events. Similar to stroke patients, in our study, a large variability in spreading depolarization responses was observed among different animals, which is probably a consequence of using CD-1 mice, a non-consanguineous strain that differs from inbred strains such as C57BL/6, and aims to model the diversity of the global human population [52]. However, our results are in agreement with the depolarization time/postischemic damage concept introduced by Dijkhuizen R.M. et al. [37], since 2-APB induced neuroprotection and concurrently shortened the total depolarization time.

A limitation of our study is attributable to the lack of specificity of 2-APB. It has been described that 2-APB is a membrane-permeable inhibitor of IP3 receptors [31,53], a recognized antagonist of Ca^2+^ entry [54,55] and an activator of transient receptor potential family channels [56]. The involvement of 2-APB in Ca^2+^ signaling has been related to its thromboprotective properties [26], and neuroprotective roles after ischemia-reperfusion [24,25]. Although the contribution of the Ca^2+^ increase in astrocytes seems dispensable for depolarization waves’ propagation [41,42], it has been reported that PIDs are associated with increased intracellular Ca^2+^ in astrocytes, as astrocytic-inositol triphosphate receptor-type-2-dependent-deficient mice display less PIDs and enhanced neuroprotection after focal ischemia [57]. Because astrocytic Ca^2+^ elevation during spreading depolarization induced by KCl (non-ischemic conditions) is insensitive to 2-APB [58], alternative directions for the therapeutic effect of this molecule should be taken. Interestingly, 2-APB is also a recognized gap junction blocker with high affinity for the neuronal gap junction Connexin-36 (Cx36) [59], which has been involved in the generation of spreading depolarization [60,61]. In photothrombotic models, the nonspecific blocker of Cx36, mefloquine, reduced neuronal death; however, this compound did not show any neuroprotective effect on Cx36-deficient mice [62], which are naturally neuroprotected from brain ischemia and show a lower incidence of spreading depolarizations [60,62]. In addition to mefloquine, other unspecific Cx36 blockers, such as quinine or quinidine, inhibit spreading depolarization [63]. In the light of this evidence, we hypothesize that the mechanism of neuroprotection exerted by 2-APB linked with a lower cumulative time of depolarization events can underlie a possible inhibition of Cx36 channels, especially because Cx36 channels are opened by ischemia [64,65] and that the neuroprotection exerted by 2-APB in our study phenocopies the decreased depolarization time observed after ischemia in Cx36-deficient mice [60].

There were several reasons to preliminary include males in our study, especially to compare our experimental design with the previous literature. Although no apparent differences have been observed in the hemodynamic responses during cortical depolarizations between male and female mice [66], a reduced threshold of cortical depolarization was reported in female mice [67]. In addition, non-specific inhibitors of TRPM-2 channels, such as 2-APB, neuroprotect male but not female animals after brain ischemia-reperfusion [25]. In contrast, similar to our study of wild-type mice treated with 2-APB, Cx36-deficient mice show a decreased depolarization time and innate neuroprotection after brain ischemia, an observation that was only determined in male animals [60]. Furthermore, previous methods developed by our group to assess at behavioral and electrophysiological levels the impact of permanent ischemia were developed in male mice [30]. Due to these premises and the controversial aspects found in the literature regarding the influence of sex due to hormonal differences (such as estrogen) in the variability of neurological responses [68], we focused this work on an analysis of one single gender to prevent a possible bias in effects non-related to the variables of study. Nevertheless, additional work can provide further information on the opportunities that 2-APB can bring to neuroprotect females after permanent ischemia.

There is a wide range of clinical and experimental evidence that supports the role of spreading depolarizations in the pathological aspects of cerebrovascular episodes such as stroke, subarachnoid hemorrhage, and traumatic brain injury and slow depolarization waves are considered one of the main contributors of secondary injury [69]. PIDs have usually been considered pharmacoresistant in the injured tissue, but 2-APB expands the short list of compounds able to target these pathological events [70]. In contrast, numerous guidelines indicate the need for rigorous preclinical studies for new neuroprotectant drugs before reaching the medical practice [71]. Thus, before undertaking clinical trials in patients, it would be advisable to cover additional aspects of molecular impact, for example, an analysis of the neuroprotective effects of 2-APB in animals with suppression or knockdown of Cx36 [60], TRPM2 [72], or TRPM7 [73], all of which are naturally protected from brain ischemia.

Although previous studies (ischemia-reperfusion) and our study (permanent ischemia) indirectly suggest that 2-APB crosses the blood–brain barrier after systemic administration, to our knowledge, there are no pharmacokinetic studies with this molecule. Previously, it has been reported that 2-APB showed neuroprotection after peripheral neuropathy caused by chemotherapy-related neurotoxicity [74]. This neuroprotection was more efficient at doses of 2 and 4 mg/kg than at higher doses (8 mg/kg), suggesting the existence of an optimal concentration window for this compound. This window will depend on every pathological scenario and should be tested in future pharmacokinetic and biodistribution assays, together with an analysis of the tolerability and long-term side effects with hematology, biochemistry, and anatomical pathology studies. The fact that PIDs can be modulated by molecules such 2-APB provides hope for future clinical studies.

## 4. Materials and Methods

### 4.1. Animals

Adult male mice (CD-1 strain, 35–45 g body weight; 3–8 months old) were bred and housed in the animal facility of the Center for Biomedical Technology in an animal room with a controlled temperature and a 12:12 light:dark cycle. Animals had free access to food and water. Animal experiments were performed according to the Animal Research: Reporting In Vivo Experiments (ARRIVE) guidelines. The number of groups and sample size analyzed in each individual study is indicated in the respective legend of every figure and listed in the following table (Table 1):

### 4.2. Permanent Middle Cerebral Artery Occlusion

Focal cerebral ischemia was induced by permanent middle cerebral artery occlusion (pMCAO) following procedures and steps previously reported by our group [30]. Briefly, mice were anesthetized with 2% isoflurane in air (21% oxygen). During anesthesia, the eyes were protected with vaseline to prevent corneal injury [75]. Under a dissection microscope, a vertical skin incision was made between the right eye and ear. The temporal muscle was separated from the skull and the right middle cerebral artery (MCA) was identified. A burr hole was made with a microdrill. The dura was resected, and the MCA was permanently ligated using a nylon suture.

### 4.3. Drug Administration

**Chart 1:**
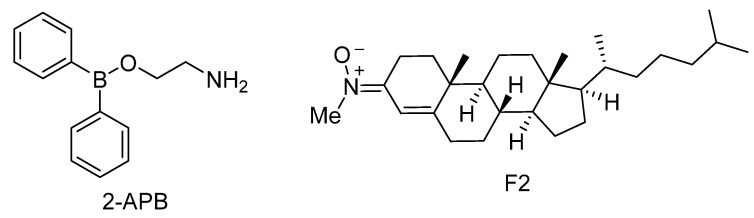
Structure of ligands 2-APB and cholesteronitrone F2.

2-APB (D9754 Sigma Aldrich) was prepared at a concentration of 1 mg/mL in 1% dimethyl sulfoxide (DMSO) in phosphate-buffered saline (PBS). For infarct size determination, behavioral and somatosensory evoked potentials (SSEPs) tests, inflammation, and neuronal death analysis, 2-APB was injected intraperitoneally (i.p.) at a dose of 7.0 mg/kg (2 doses were applied at 2 and 10 h after pMCAO). For cortical spreading depolarization (CSD) studies, 2-APB was injected i.p. at the same dose (7 mg/kg) 1 h before starting the CSD recordings. For oxidative stress outcomes, a single dose of 2-APB was injected i.p. (7 mg/kg) 2 h after pMCAO. In all the studies, a control group received an equivalent volume of 1% DMSO (untreated vehicle group). Cholesteronitrone F2 was prepared at a concentration of 0.02 mg/mL in 10% ethanol in PBS. For oxidative stress assessment, one dose of F2 (0.1 mg/kg) was injected i.p. at 2 h after pMCAO while for neuronal death analysis, 2 doses (0.1 mg/kg) were i.p. injected at 2 and 10 h after pMCAO. The chemical structures of 2-APB and F2 are illustrated in Chart 1.

### 4.4. Infarct Volume and Area Measurement

The infarct size was evaluated as described elsewhere [30]. Mice were sacrificed by cervical dislocation 24 h after pMCAO. The brains were cut into 1-mm-thick coronal slices using a brain matrix (WPI, Sarasota, FL, USA). The sections were immersed for 10 min in a tempered solution of 2,3,5-triphenyltetrazolium (TTC, T8877, Sigma Aldrich, St. Louis, MO, USA) in PBS to stain live cells that contained functional mitochondria. Brain slices were fixed with 4% paraformaldehyde (PFA) in PBS. Coronal sections were digitalized, and the infarct size was determined with the image processing and analysis program ImageJ (NIH). Infarct volume percentages were calculated with respect to the volume of the contralateral hemisphere.

### 4.5. Behavioral Tests

Functional outcome after 2-APB administration was examined with the cylinder and grid walking tests one week after stroke [30]. To evaluate forepaw asymmetry, mice were placed in a clear Plexiglas cylinder. During normal vertical exploration, a minimum of 25 contacts were counted and recorded per trial, and the forepaws (right, left, or both) used for each contact were scored. The laterality index (LI) was scored as LI = [Contacts (Right) − Contacts (Left)]/[Contacts (Right) + Contacts (Left) + Contacts (both)]. In the grid walking test, mice were allowed to freely move over a metal grid with 13 × 13 mm^2^ squares for 3 min. The performance of each animal was videotaped, and the total numbers of right and left forepaw steps during spontaneous locomotion (typical range of 200–400 total steps) and the total number of foot faults for each forelimb were scored. Untreated and 2-APB-treated animals underwent behavioral tests before and one week after pMCAO. In both functional tests, video recordings were blindly evaluated by R. F-S.

### 4.6. Evaluation of Functional Cortical Territory

SSEPs recordings were performed as we previously described [30]. To examine the functionality of the cortical territory, SSEPs were longitudinally recorded in both hemispheres prior to and after unilateral stroke (pMCAO) in untreated vehicle and 2-APB-treated mice (72 h, 1, 2, and 3 weeks after stroke). For SSEPs recordings, two electrodes were placed in the right and left somatosensory cortices, respectively (±2.0 mm lateral and +0.5 mm rostrocaudal from bregma). Two additional electrodes were implanted over the visual cortex and served as the indifferent and ground electrodes (±2.0 mm lateral and −3 mm rostrocaudal from bregma). The electrodes were secured in place with dental acrylate (Duraly, IL, USA). Extreme care was observed to prevent contact between dental acrylate and the temporal bone, which was essential for the opening of a craniotomy window in this area for pMCAO. Animals were allowed to recover for a minimum of 7 d before starting the recording sessions and the subsequent pMCAO surgery. In every recording session, contralateral and ipsilateral potentials were obtained for each hemisphere in response to forepaw electrical stimulation (1–2 mA, a current level to produce a supramaximal motor response). Cortical field potentials were amplified (×10^3^) and filtered (bandpass, 10–2000 Hz) using a portable electromyography (EMG)-evoked potential (EP) device (Micromed, Mogliano Veneto, Treviso, Italy).

### 4.7. Cortical Depolarization Recordings

Spontaneous waves of depolarization were recorded in response to pMCAO (ischemic animals). Craniotomies were made by stereotaxic surgery as previously described with some modifications [30]. Animals under anesthesia were secured with ear bars in a stereotaxic frame (David Kopf Instruments, Tujunga, CA, USA). The depth of anesthesia was assessed by the loss of tail reflex after pinching. The body temperature was maintained close to 37 °C using a heating pad. Under aseptic conditions, a skin incision was carefully made, and two small holes were drilled through the skull of the right hemisphere. The craniotomies were performed at parietal (2.3 mm lateral and −0.10 mm rostrocaudal) and occipital (2.3 mm lateral and −4.0 mm rostrocaudal) cortices for direct current (DC) potential recordings. Considering the pMCAO model, the parietal craniotomy was anatomically localized in areas close to the perilesional border, according to previous experience with this specific mouse strain [44]. DC potential shifts were recorded under anesthesia with urethane (1.8 g/kg) at parietal and occipital cortices (~300 mm in depth) with Ag/AgCl wire electrodes inserted into glass micropipettes filled with 150 mM NaCl. Micropipettes were fabricated using a micropipette puller (P-97, Sutter Instruments, Novato, CA, USA) with impedances ranging from 1–2 MΩ. A third Ag/AgCl reference electrode was inserted subcutaneously into the neck. Extracellular signals were amplified (×10) and filtered (0–100 Hz) with a DAM50 amplifier (World Precision Instruments, Sarasota, FL, USA). Signals were digitalized and acquired with LabVIEW Biomedical Toolkit software (National Instruments, Austin, TX, USA).

### 4.8. Cerebral Blood Flow Assessment

In some animals, local cerebral blood flow (CBF) was measured with a laser-Doppler flow probe (Periflux System 5000, Perimed, Järfälla, Sweden) while the DC signal was simultaneously recorded. The Doppler probe was placed over the intact skull adjacent to the parietal cortex electrodes (2.3 mm lateral and −2.6 mm rostrocaudal). Blood flow changes were analyzed with PSW2 software (Perimed) and expressed as the percentage of change. Further analysis on percentage changes was carried out using Clampfit software (v10.7).

### 4.9. Oxidative Stress Measurement

In vivo determination of oxidative stress in perilesional areas was performed as previously described with several modifications [76]. Four hours after pMCAO, mice were intravenously injected with 200 mL of dihydroetidium (DHE, 37291, Sigma Aldrich) at a concentration of 1 mg/mL in 5% DMSO in PBS. Then, 4 h later, the mice were transcardially perfused with PBS under terminal anesthesia. The brains were removed and fixed overnight in 4% PFA. The brains were then dehydrated consecutively in 10 and 30% sucrose solutions and cut into 10-mm-thick coronal sections in a freezing microtome and mounted on gelatin-coated slides. Nuclei were stained with DAPI (F6057, Sigma Aldrich). The DHE signal was analyzed under a fluorescence microscope (Leica DMI3000, Nussloch, Germany). The DHE signal was quantified with imageJ (NIH) and normalized to the number of nuclei in the medial cortex and striatum regions and expressed as a percentage with respect to the untreated vehicle control group.

### 4.10. Immunohistochemistry

For the quantification of inflammatory cells present in perilesional areas, 72 h after the ischemia, mice were transcardially perfused with PBS and 4% PFA under terminal anesthesia. The brains were dehydrated as previously described and cut into 30-μm-thick coronal sections in a freezing microtome. Sections were incubated with primary antibodies (rat anti-GFAP, 1:800, 13-0300, Invitrogen; and rabbit anti-Iba1, 1:1000, ab178847, Abcam) and with fluorescently labeled secondary antibodies (donkey anti-rat, 1:800, 712-546-153, Jackson ImmunoResearch; and goat anti-rabbit, 1:800, 111-165-003, Jackson ImmunoResearch). Nuclei were stained with Hoechst 33342 and the sections were mounted on gelatin-coated slides. Perilesional areas were photographed under a fluorescence microscope (Leica DMI3000, Nussloch, Germany). The number of GFAP-positive and Iba1-positive cells was analyzed using ImageJ (NIH) and normalized to the number of nuclei.

### 4.11. TUNEL Assay

Apoptotic neurons within brain sections were detected using the Terminal deoxynucleotidyl transferase-mediated dUTP Nick-End Labeling (TUNEL) assay (Promega). Brains used for immunohistochemical analysis were also cut into coronal sections (5 μm-thick) in a freezing cryostat and prepared on Real Capillary Gap microscope slides (K8020, Dako). Sections were post-fixed, permeabilized, and processed for TUNEL assay as previously described [35]. Briefly, brain sections were incubated with terminal deoxynucleotidyl transferase (TdT) and fluorescein-12-dUTP for 1.5 h at 37 °C, as described by the supplier, and the reaction was stopped by washing in saline-sodium citrate buffer. After washing in PBS, the sections were then mounted with coverslips in an antifade solution, with glycerol-buffer containing 4.6 mM p-phenylenediamine and 30 mM bisbenzimide (Hoechst 33342), for nuclear staining. Fields of the primary somatosensory cortex were randomly selected in the ipsilateral region from a given brain section with a fluorescence microscope (40× objective), and the selected images were digitized with a color CCD camera. The green fluorescence intensity of the TUNEL labeling nuclei was evaluated and normalized to the number of Hoechst staining nuclei using an image analysis software (ImageJ, NIH). At least four images per brain sample, and 3 to 7 brains per experimental group, were evaluated and averaged; treatment information was kept concealed throughout the study.

### 4.12. Statistical Analysis

SigmaPlot (Systat, Germany) was used for the statistical analyses. All data are expressed as the mean ± the standard error of the mean (SEM). To examine significant differences in infarct volume, oxidative stress, and inflammation a Student’s t-test analysis was applied. The infarct area along the rostrocaudal axis, sensorimotor function (cylinder and grid walking tests), and cortical somatosensory activity (SSEPs) in the time after treatment were analyzed using two-way analysis of variance (ANOVA), followed by Tukey’s post hoc test. Two-way ANOVA was performed using infarct areas, deficits, and voltage amplitudes as dependent variables and distance from bregma or time and groups (vehicle and 2-APB) as independent variables. Neuronal death was analyzed using the Kruskal–Wallis test followed by the Mann–Whitney test. *p*-values below 0.05 were considered statistically significant.

## 5. Conclusions

Collectively, we provide data on the neuroprotective properties of 2-APB in a mouse model of permanent ischemia. The neuroprotection exerted by 2-APB was not due to a possible antioxidant effect, a property that was ascribed for the first time to the cholesteronitrone F2 in this specific permanent ischemic model. The effects of 2-APB were mainly linked to shortening of PIDs while residual cerebral blood perfusion in penumbra remained higher than in untreated stroke animals. Although it is conceivable that, especially after ischemia-reperfusion, the therapeutic effect of 2-APB can be linked with prevention of harmful Ca^2+^ entry in cerebral and non-cerebral pathologies, to our knowledge, the causal link between 2-APB and spreading depolarization has not previously been reported in vivo in the context of PIDs waves generated during brain ischemia. Whether this latter effect is only intensified after prolonged ischemia is unknown since the effect of 2-APB on SD in ischemia-reperfusion models has never been examined. SD waves and PIDs have been found in patients with brain injury and probably constitute the most relevant mechanism for the expansion of the infarcted core [45]. Pharmacological modulation of post-ischemia SD and PIDs opens alternative avenues to drive neuroprotection after brain stroke.

## Figures and Tables

**Table 1:** Total number of animals and distribution between groups and experiments.

Studies (*)	Groups	Animals (n)
Infarct volume and area determination	Vehicle	8
	2-APB	9
Behavioral testing	Vehicle	14 (gwt), 8 (ct)
	2-APB	15 (gwt), 11 (ct)
Somatosensory evoked potentials recordings	Vehicle	7
	2-APB	11
Oxidative stress, inflammation, and neuronal death studies	Vehicle	8 (os), 7 (inf and nd)
	2-APB	8 (os), 7 (inf and nd)
	F2	5 (os), 3 (nd)
Peri-infarct depolarizations recordings	Vehicle	16
	2-APB	11
Cerebral blood flow assessments	No stroke	3
	Sham	5
	Vehicle	8
	2-APB	8

* A total of 172 mice were used in this study. Animal groups were classified as non-treated (administration of vehicle) or treated (administration of 2-APB or F2) mice. For cerebral blood flow determination, two additional groups were included: no stroke (non-operated animals) and sham (operated animals subjected to an identical procedure to stroke animals with the exception that the middle cerebral artery was not ligated). Abbreviations; gwt: grid walking test; ct: cylinder test.; os: oxidative stress; inf: inflammation; nd: neuronal death.

## Data Availability

Not applicable.

## Supplementary Materials

The following supporting information can be downloaded at: www.mdpi.com/article/10.3390/ijms23137449/s1.
