# Supplementary material for: Postischemic Neuroprotection of Aminoethoxydiphenyl Borate Associates Shortening of Peri-Infarct Depolarizations
Source: Int J Mol Sci. 2022 Jul 4;23(13):7449. doi: 10.3390/ijms23137449 (PMC9266990; doi:10.3390/ijms23137449)
Supplement: Supplementary file 1 [file ijms-23-07449-s001.zip › ijms-1767518-supplementary.pdf]

SUPPLEMENTARY MATERIAL

Postischemic Neuroprotection of Aminoethoxydiphenyl Borate Associates Shortening of Peri-Infarct Depolarizations

Rocío Fernández-Serra, Emma Martínez-Alonso, Alberto Alcázar, Mourad Chioua, José Marco-Contelles, Ricardo Martínez-Murillo, Milagros Ramos, Gustavo V. Guinea and Daniel González-Nieto

Supplementary material consists of:

1. Supplementary Figures:

♦ Supplementary Figure S1- Evaluation of the functional somatosensory cortical territory in 2-APB treated mice at 2 and 3 weeks post-ischemia

♦ Supplementary Figure S2- Analysis of the inflammation area

♦ Supplementary Figure S3- DC shift components

♦ Supplementary Figure S4- Characterization of hypoperfusion-hyperemia events

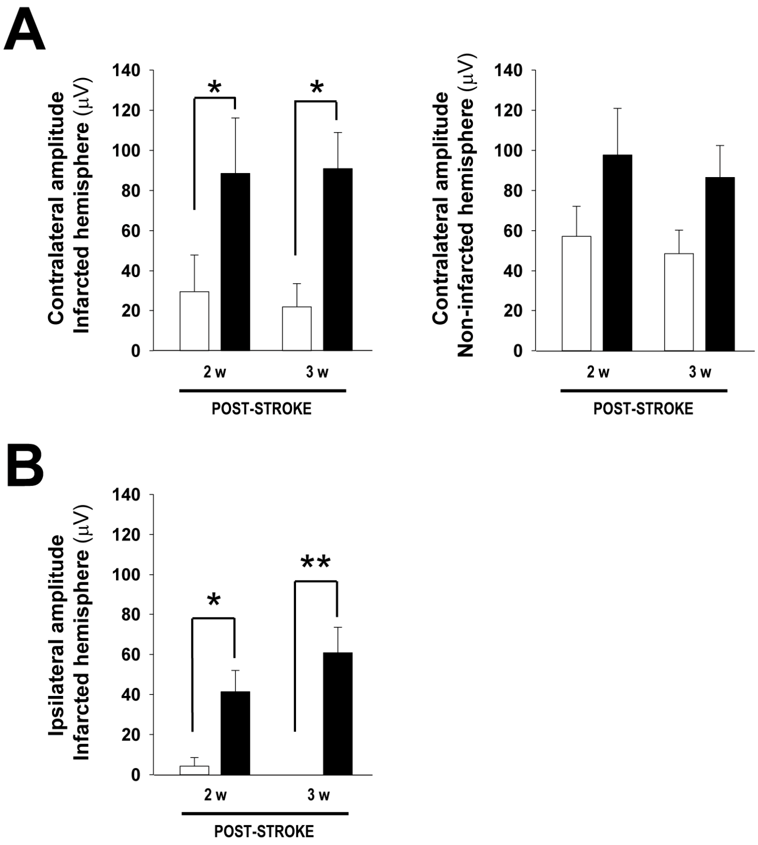

Supplementary Figure S1. Evaluation of the functional somatosensory cortical territory in untreated and 2-APB treated mice at 2- and 3-weeks post-ischemia. (A) Amplitude of contralateral SSEPs recorded in the right (infarcted) and left (non-infarcted) hemispheres at 2 (2w) and 3 (3w) weeks respectively after stroke. (B) Amplitude of ipsilateral SSEPs in the left hemisphere at 2 and 3 weeks after stroke. A minimum of 5 mice were analyzed per group. The data are shown as the means ± SEM. The asterisks denote significant differences between groups (two way ANOVA; $*p<0.05$, $**p<0.01$).

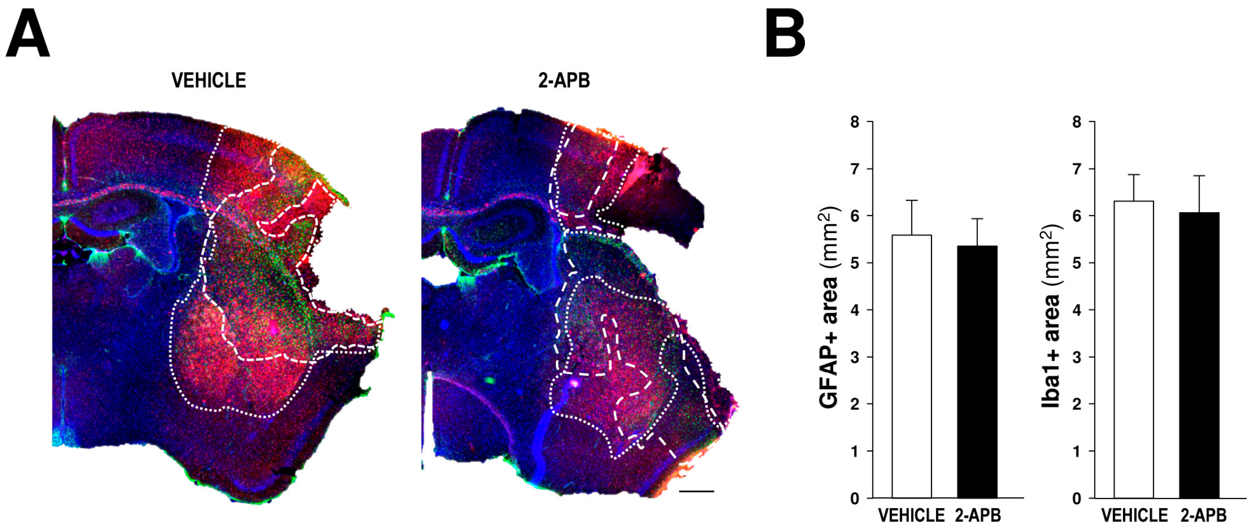

Supplementary Figure S2. Analysis of the inflammation area. (A) Representative fluorescent microscopy images showing how GFAP-positive (dashed line) and Iba1-positive (dotted line) areas were quantified. Scale bar: 500 µm. (B) Quantification of the area occupied by GFAP-positive (left) and Iba1-positive cells. Four images were analyzed per brain from 7 mice per group (vehicle and 2-APB treated mice).

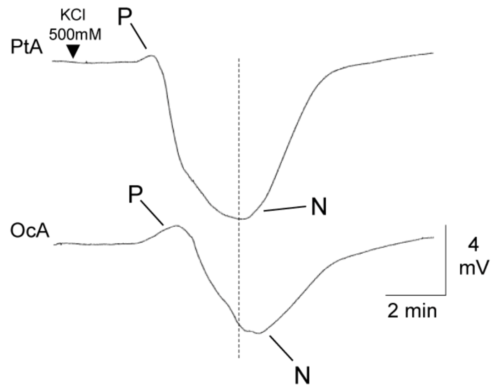

Supplementary Figure S3. DC shift components. Representative traces of DC shifts elicited by the application of KCl to the frontal cortex. Every DC shift shows several wave components that propagate from parietal (PtA) to occipital (OcA) cortices.

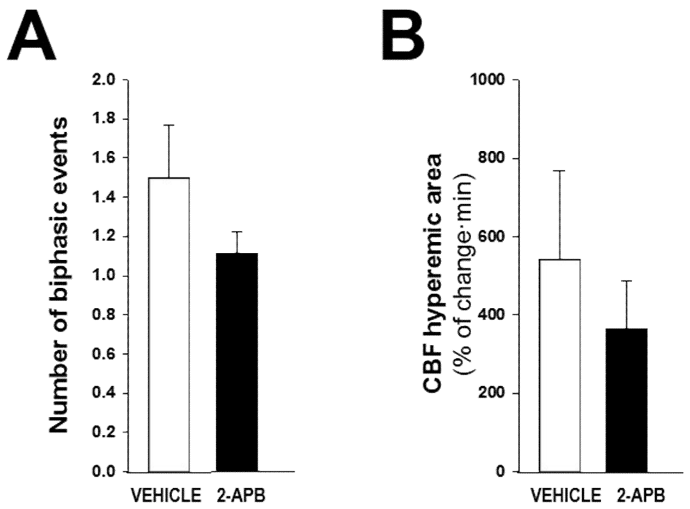

Supplementary Figure S4. Analysis of hypoperfusion-hyperemia events. (A) Quantification of the number of hypoperfusion-hyperemic events elicited after pMCAO. (B) Total hyperemic area in 100 min of CBF recordings. A total of 8 mice per group, untreated (vehicle) and 2-APB-treated mice, were analyzed.
